# Association between dietary mineral intakes and urine flow rate: data from the 2009–2018 National Health and Nutrition Examination Survey

**DOI:** 10.3389/fnut.2024.1424651

**Published:** 2024-09-18

**Authors:** Ming Li, Jiqian Zhang, Jiasen Ding, Zhan Gao

**Affiliations:** ^1^Graduate School, Beijing University of Chinese Medicine, Beijing, China; ^2^Xiyuan Hospital, China Academy of Chinese Medical Sciences, Beijing, China

**Keywords:** urine flow rate, intakes of dietary minerals, dietary mineral intakes, cross-section study, NHANES

## Abstract

**Background:**

Minerals play an important role in human health, but their effect on urinary function remains controversial. The aim of this study was to assess the association between dietary intake of minerals (Ca, P, Mg, Fe, Zn, Cu, Na, K, Se) and urine flow rate (UFR).

**Methods:**

We conducted a cross-sectional study using the National Health and Nutrition Examination Survey (NHANES, 2009–2018) database. Multivariate regression and smooth curve fitting were used to investigate the association between dietary mineral intakes and UFR. Subgroup analyses and interaction tests were used to investigate whether this association was stable in the population.

**Results:**

Our study involving 10,229 representative adult NHANES participants showed an association between Mg intake and UFR in a linear regression model for continuous variables. And in the model analysis of tertile categorical variables, we observed a positive association between six mineral intakes (Ca, Mg, Zn, Cu, Na, and K) and UFR. Smoothed curve fitting and threshold effect analysis further support the nonlinear relationship between mineral intakes and UFR. Subgroup analyses and interaction tests ensured the reliability and robustness of the findings.

**Conclusion:**

This study examined the effects of nine dietary minerals on UFR and found that intake of Ca, Mg, Zn, Cu, Na, and K were positively correlated with UFR, suggesting that these minerals may have a positive effect on improving urinary function. In particular, Mg showed a more significant positive correlation with UFR in women, while Na showed a stronger positive correlation in diabetics. However, P, Fe and Se did not show significant correlations. In summary, although these findings provide a preliminary understanding of the relationship between dietary minerals and urinary function, further prospective studies are still necessary to validate these relationships and explore the physiologic mechanisms underlying them.

## 1 Introduction

Voiding dysfunction is a dysfunction of the bladder, urethra, and their associated nervous and muscular systems that results in an abnormality in the normal process of urination, and is the most common symptom seen in urologic clinics, especially in the elderly (1, 2). It is estimated that by 2025, more than 42 million people in the United States will experience urinary dysfunction (3). Difficulty in voiding significantly reduces the quality of life for patients and leads to serious economic and public health problems (4, 5). Urine flow rate (UFR) is a measurement of the amount of urine produced over a specific period of time and can provide some assessment of voiding function (6, 7). A daily urine output of less than 400 mL is referred to as oliguria and is the minimum amount of urine required to eliminate metabolic waste (8). Healthy eating patterns play an important role in the prevention and treatment of many chronic diseases (9–11), although dietary minerals do not make up a large proportion of total dietary intake, they play a very important role in health (12). Several studies have reported that inadequate mineral intake is associated with blood pressure and abnormalities in lipid metabolism and glucose metabolism (13–15). Many studies have reported dietary associations with voiding function (16–18), but only few have examined the correlation between dietary minerals and voiding function (19). Therefore, the aim of this study was to investigate the relationship between dietary intake of nine common minerals [calcium (Ca), phosphorus (P), magnesium (Mg), iron (Fe), zinc (Zn), copper (Cu), sodium (Na), potassium (K) and selenium (Se)] and UFR. We hope that this study will provide some information for risk management and preventive interventions for patients with urinary dysfunction.

## 2 Methods

### 2.1 Study design and participants

**Figure 1:**
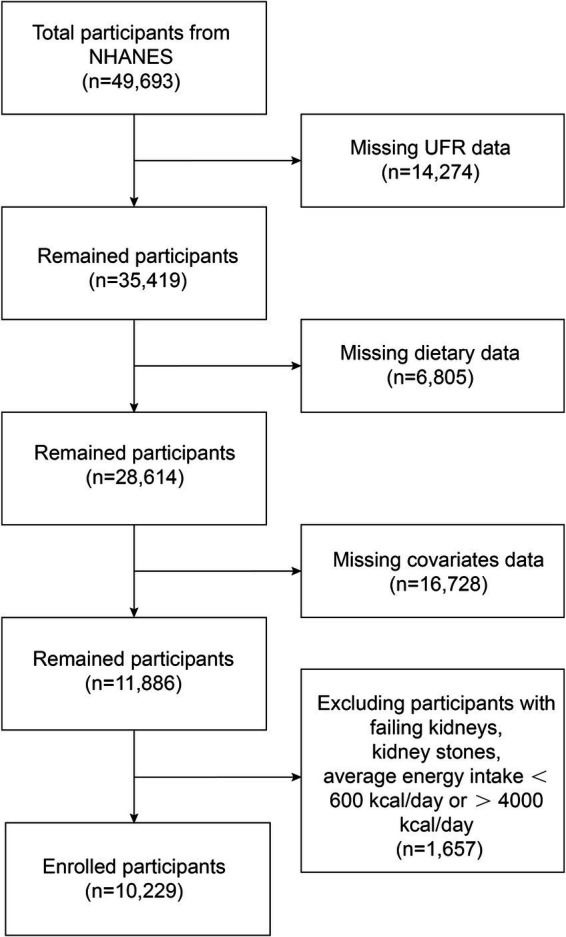
The flowchart of participants.

NHANES data from five cycles (2009–2010, 2011–2012, 2013–2014, 2015–2016, and 2017–2018) were used in this study. Figure 1 shows a flowchart of the participant recruitment process. There were a total of 49,693 participants over the five cycles, and we first excluded 14,274 participants with missing UFR, then 6,805 with missing dietary mineral intake, 16,728 with missing covariates data, finally 1,657 participants with failing kidneys, kidney stones, and average energy intake below 600 kcal/day or above 4,000 kcal/day were excluded. Ultimately, a total of 10,229 participants were included in this study. The NHANES study was conducted with the approval of the Ethics Review Committee of the National Center for Health Statistics, and it met the ethical standards of the 1964 Declaration of Helsinki and its subsequent amendments, with written informed consent signed by all participants.

### 2.2 Measurement of UFR

Participants will be asked to record the time of their last urination prior to coming to the mobile examination center (MEC), and then, record the time and volume of urine samples collected while the participant is at the MEC and calculate the UFR based on the volume of the urine samples collected as well as the duration of time that elapsed between urinations. To ensure sufficient urine volume for analysis, each participant was allowed to provide up to three urine samples. The formula for calculating UFR was: UFR = (total urine volume)/(total time duration). Detailed instructions for urine sample collection and processing can be found in the NHANES Laboratory Procedures Manual (LPM).

### 2.3 Intake of dietary minerals

The NHANES dietary intake data are the types and amounts of food and beverages (including all types of water) consumed by participants in the 24 h prior to the interview (midnight to midnight), and estimates of energy, nutrient, and other food components consumed from these foods and beverages. All participants underwent two 24-h dietary recall interviews, the first dietary recall interview was collected in person at the MEC, and the second interview was collected by telephone 3 to 10 days later. Dietary mineral intakes were derived by calculating the average of the two interview data.

### 2.4 Covariates

A number of covariates were included in this study, including age (years), gender (male/female), race (Mexican American, non-Hispanic Black, non-Hispanic White and other race), education level (lower than high school, high school or equivalent and college or above), income-to-poverty ratio (PIR), body mass index (BMI), serum creatinine (SCr), energy intake, protein intake, carbohydrate intake, total sugars intake, total fat intake, smokers (yes/no), alcohol drinkers (yes/no), hypertension (yes/no), diabetes (yes/no), history of cardiovascular diseases (CVD). BMI was calculated by dividing weight (in kilograms) by the square of height (in meters) and was divided into 4 categories: underweight (< 18.50 kg/m^2^), normal weight (18.5 kg/m^2^ ≤ BMI < 25.0 kg/m^2^), overweight (25 kg/m^2^ ≤ BMI < 30.00 kg/m^2^) and obese (≥ 30.0 kg/m^2^). History of CVD included congestive heart failure, coronary heart disease, angina pectoris, heart attack, and stroke.

### 2.5 Statistical analysis

Participant demographic data were assessed through chi-square and t-tests for gender categorization. Weighted multiple regression analyses were used to test the linear relationship between dietary mineral intakes and UFR. After transforming dietary mineral intakes from continuous variables to categorical variables (tertiles), a trend test was used to examine the trend of linear association between dietary mineral intakes and UFR. To explore the relationship between dietary mineral intakes and UFR, subgroup analyses were conducted across variables such as gender, BMI, hypertension, and diabetes to assess the consistency of these associations. Additionally, interaction tests were utilized to further examine the uniformity of these relationships across the different subgroups. Nonlinear association between dietary mineral intakes and UFR were investigated using smoothed curve fitting. All statistical analyses were conducted using R (version 4.3.0) and EmpowerStats (version 5.0), with a significance threshold set at a two-sided *p*-value of less than 0.05.

## 3 Results

### 3.1 Baseline characteristics

A total of 10,229 individuals with complete survey data were enrolled in this study, including 5,075 males and 5,154 females, and the mean age of the subjects was 46.80 ± 16.95 years, Table 1 shows the baseline characteristics of the subjects. The following variables showed statistically significant differences between men and women: age, race, education, BMI, SCr, energy intake, protein intake, carbohydrate intake, total sugars intake, total fat intake, smokers, alcohol drinkers, hypertension, diabetes, history of CVD, dietary mineral intakes (Ca, P, Mg, Fe, Zn, Cu, Na, K, Se), and UFR (all *p* < 0.05).

**Table 1:** The characteristics of participants.

Characteristics	Male	Female	ALL	*p*-value
	*N* = 5,075	*N* = 5,154	*N* = 10,229	
Age (years)	47.34 ± 17.09	46.27 ± 16.80	46.80 ± 16.95	0.002
Race/ethnicity, (%)				<0.001
Non-Hispanic White	718 (14.15%)	598 (11.60%)	1,316 (12.87%)	
Non-Hispanic Black	991 (19.53%)	1,081 (20.97%)	2072 (20.26%)	
Mexican American	2,221 (43.76%)	2,404 (46.64%)	4,625 (45.21%)	
Other races	1,145 (22.56%)	1,071 (20.78%)	2,216 (21.66%)	
Education level, (%)				<0.001
< high school	911 (17.95%)	697 (13.52%)	1,608 (15.72%)	
High school	1,148 (22.62%)	1,034 (20.06%)	2,182 (21.33%)	
> high school	3,016 (59.43%)	3,423 (66.41%)	6,439 (62.95%)	
Family PIR	2.81 ± 1.66	2.74 ± 1.66	2.77 ± 1.66	0.036
BMI, (%)				<0.001
<18.5	55 (1.08%)	81 (1.57%)	136 (1.33%)	
≥18.5, <25	1,308 (25.77%)	1,582 (30.69%)	2,890 (28.25%)	
≥25, <30	1,907 (37.58%)	1,435 (27.84%)	3,342 (32.67%)	
≥30	1,805 (35.57%)	2,056 (39.89%)	3,861 (37.75%)	
Smokers, (%)				<0.001
Yes	2,677 (52.75%)	2,007 (38.94%)	4,684 (45.79%)	
No	2,398 (47.25%)	3,147 (61.06%)	5,545 (54.21%)	
Alcohol drinkers, (%)				<0.001
Yes	154 (3.03%)	29 (0.56%)	183 (1.79%)	
No	4,921 (96.97%)	5,125 (99.44%)	10,046 (98.21%)	
Diabetes, (%)				<0.001
Yes	791 (15.59%)	612 (11.87%)	1,403 (13.72%)	
No	4,284 (84.41%)	4,542 (88.13%)	8,826 (86.28%)	
Hypertension, (%)				<0.001
Yes	2,038 (40.16%)	1,816 (35.23%)	3,854 (37.68%)	
No	3,037 (59.84%)	3,338 (64.77%)	6,375 (62.32%)	
History of CVD, (%)				<0.001
Yes	461 (9.08%)	311 (6.03%)	772 (7.55%)	
No	4,614 (90.92%)	4,843 (93.97%)	9,457 (92.45%)	
SCr (mg/dL)	0.98 ± 0.21	0.76 ± 0.18	0.87 ± 0.23	<0.001
Energy (kcal/day)	2308.62 ± 697.19	1811.34 ± 586.23	2058.06 ± 690.00	<0.001
Protein (gm/day)	93.17 ± 33.65	70.01 ± 25.32	81.50 ± 31.92	<0.001
Carbohydrate (gm/day)	268.95 ± 94.45	218.61 ± 80.03	243.58 ± 91.03	<0.001
Total sugars (gm/day)	112.11 ± 61.63	97.06 ± 50.69	104.53 ± 56.88	<0.001
Total fat (gm/day)	86.96 ± 34.36	70.24 ± 29.11	78.54 ± 32.90	<0.001
Ca (mg/day)	1000.09 ± 484.86	848.36 ± 392.88	923.64 ± 447.38	<0.001
P (mg/day)	1521.87 ± 535.18	1188.71 ± 417.74	1354.00 ± 507.70	<0.001
Mg (mg/day)	329.12 ± 131.43	269.31 ± 104.10	298.99 ± 122.16	<0.001
Fe (mg/day)	16.11 ± 7.50	12.63 ± 5.70	14.36 ± 6.88	<0.001
Zn (mg/day)	12.68 ± 6.30	9.47 ± 4.61	11.07 ± 5.74	<0.001
Cu (mg/day)	1.35 ± 0.78	1.13 ± 0.67	1.24 ± 0.73	<0.001
Na (mg/day)	3898.40 ± 1385.67	3005.21 ± 1109.37	3448.35 ± 1331.18	<0.001
K (mg/day)	2903.29 ± 1067.98	2359.87 ± 848.95	2629.48 ± 1001.38	<0.001
Se (mcg/day)	130.78 ± 51.51	98.25 ± 39.40	114.39 ± 48.61	<0.001
UFR (mL/h)	66.70 ± 56.06	62.99 ± 62.08	64.83 ± 59.19	<0.001

Mean ± SD for continuous variables: the *p* value was calculated by the weighted linear regression model; (%) for categorical variables: the *p* value was calculated by the weighted chi-square test.

### 3.2 Association between dietary mineral intakes and UFR

Table 2 demonstrates the results of weighted generalized linear regression models based on different adjusted covariates with transformation treatment of dietary mineral intake from continuous to tertile categorical variables to assess the association between dietary mineral intake and UFR. In the linear regression models for continuous variables, both the unadjusted and partially adjusted models (Model I) showed a strong positive association between total dietary mineral intake and UFR at a significance level (all *p* < 0.0001). However, in the fully adjusted model (Model II), only Mg remained significant (*p* < 0.0001), while the *p*-values for the rest of the dietary minerals exceeded 0.05 and did not show statistical significance.

**Table 2:** Associations between dietary mineral intakes and UFR.

Exposure	Crude model	Model I	Model II
	β (95% CI) *p*-value	β (95% CI) *p*-value	β (95% CI) *p*-value
Ca (continuous)	0.01 (0.01, 0.01) <0.0001	0.01 (0.01, 0.01) <0.0001	0.00 (−0.01, 0.01) 0.8851
Ca (categories)			
Tertile 1	Reference	Reference	Reference
Tertile 2	7.20 (3.53, 10.86) 0.0001	5.82 (2.15, 9.49) 0.0019	6.15 (1.97, 10.33) 0.0039
Tertile 3	15.83 (12.28, 19.39) <0.0001	13.37 (9.72, 17.02) <0.0001	15.45 (9.04, 21.85) <0.0001
*P* for trend	<0.0001	<0.0001	<0.0001
P (continuous)	0.01 (0.01, 0.02) <0.0001	0.01 (0.01, 0.01) <0.0001	0.00 (−0.01, 0.01) 0.7813
P (categories)			
Tertile 1	Reference	Reference	Reference
Tertile 2	11.86 (8.23, 15.48) <0.0001	10.87 (7.21, 14.52) <0.0001	6.19 (1.78, 10.60) 0.0060
Tertile 3	14.52 (10.97, 18.08) <0.0001	13.11 (9.32, 16.90) <0.0001	0.86 (−6.20, 7.92) 0.8114
*P* for trend	<0.0001	<0.0001	0.9438
Mg (continuous)	0.07 (0.06, 0.09) <0.0001	0.07 (0.06, 0.08) <0.0001	0.05 (0.03, 0.08) <0.0001
Mg (categories)			
Tertile 1	Reference	Reference	Reference
Tertile 2	9.53 (5.92, 13.14) <0.0001	8.74 (5.09, 12.39) <0.0001	6.34 (2.12, 10.57) 0.0032
Tertile 3	23.99 (20.44, 27.55) <0.0001	23.14 (19.41, 26.86) <0.0001	16.97 (10.59, 23.35) <0.0001
*P* for trend	<0.0001	<0.0001	<0.0001
Fe (continuous)	0.65 (0.44, 0.86) <0.0001	0.57 (0.35, 0.79) <0.0001	−0.18 (−0.52, 0.16) 0.3088
Fe (categories)			
Tertile 1	Reference	Reference	Reference
Tertile 2	9.30 (5.73, 12.87) <0.0001	8.25 (4.65, 11.84) <0.0001	4.98 (0.84, 9.12) 0.0184
Tertile 3	12.05 (8.49, 15.61) <0.0001	10.79 (7.08, 14.50) <0.0001	4.34 (−1.88, 10.56) 0.1718
*P* for trend	<0.0001	<0.0001	0.2242
Zn (continuous)	0.70 (0.45, 0.96) <0.0001	0.59 (0.32, 0.86) <0.0001	−0.18 (−0.60, 0.24) 0.4082
Zn (categories)			
Tertile 1	Reference	Reference	Reference
Tertile 2	10.64 (7.04, 14.25) <0.0001	9.31 (5.67, 12.95) <0.0001	6.75 (2.64, 10.85) 0.0013
Tertile 3	13.15 (9.59, 16.71) <0.0001	11.75 (7.96, 15.54) <0.0001	7.27 (1.24, 13.30) 0.0182
*P* for trend	<0.0001	<0.0001	0.0332
Cu (continuous)	7.52 (5.70, 9.34) <0.0001	6.73 (4.87, 8.59) <0.0001	1.33 (−1.06, 3.72) 0.2749
Cu (categories)			
Tertile 1	Reference	Reference	Reference
Tertile 2	10.67 (7.08, 14.27) <0.0001	9.95 (6.33, 13.56) <0.0001	8.04 (3.99, 12.10) 0.0001
Tertile 3	24.26 (20.73, 27.79) <0.0001	22.94 (19.27, 26.60) <0.0001	18.50 (12.94, 24.07) <0.0001
*P* for trend	<0.0001	<0.0001	<0.0001
Na (continuous)	0.00 (0.00, 0.00) <0.0001	0.00 (0.00, 0.00) <0.0001	0.00 (−0.00, 0.00) 0.5913
Na (categories)			
Tertile 1	Reference	Reference	Reference
Tertile 2	8.39 (4.80, 11.98) <0.0001	7.48 (3.84, 11.12) <0.0001	6.91 (2.52, 11.30) 0.0020
Tertile 3	11.84 (8.31, 15.38) <0.0001	10.75 (6.93, 14.56) <0.0001	8.35 (1.26, 15.44) 0.0210
*P* for trend	<0.0001	<0.0001	0.0220
K (continuous)	0.01 (0.01, 0.01) <0.0001	0.01 (0.01, 0.01) <0.0001	0.00 (−0.00, 0.00) 0.1948
K (categories)			
Tertile 1	Reference	Reference	Reference
Tertile 2	9.50 (5.89, 13.10) <0.0001	8.98 (5.33, 12.62) <0.0001	7.40 (3.14, 11.66) 0.0007
Tertile 3	20.34 (16.79, 23.90) <0.0001	19.78 (16.03, 23.53) <0.0001	15.78 (9.32, 22.23) <0.0001
*P* for trend	<0.0001	<0.0001	<0.0001
Se (continuous)	0.10 (0.07, 0.13) <0.0001	0.10 (0.07, 0.13) <0.0001	0.03 (−0.03, 0.09) 0.2699
Se (categories)			
Tertile 1	Reference	Reference	Reference
Tertile 2	8.86 (5.31, 12.41) <0.0001	7.87 (4.28, 11.47) <0.0001	3.59 (−0.68, 7.87) 0.0992
Tertile 3	10.66 (7.09, 14.22) <0.0001	9.85 (6.03, 13.67) <0.0001	−0.34 (−7.17, 6.49) 0.9225
*P* for trend	<0.0001	<0.0001	0.8831

Crude model: no covariates were adjusted. Model I: age, gender, race, and education level were adjusted. Model II: age, gender, race, education level, PIR, BMI, SCr, energy intake, protein intake, carbohydrate intake, total sugars intake, total fat intake, smokers, alcohol drinkers, hypertension, diabetes, history of CVD, and mineral intakes (Ca, P, Mg, Fe, Zn, Cu, Na, K, Se) were adjusted.

In the model analysis of tertile categorical variables, the unadjusted and partially adjusted models also showed significance levels of *p* < 0.05 for all dietary mineral intakes. In the fully adjusted model (Model II), the significance of Mg remained unchanged (*p* < 0.001), and the Ca, Zn, Cu, Na, K in the tertile categories showed statistical significance as well (*p* < 0.05). The results show that there is a significant linear relationship between the Ca, Mg, Zn, Cu, Na, K tertiles and UFR (all *P* for trend <0.05). In the fully adjusted model (Model II), the *β*-values for the highest tertile (Tertile 3) compared to the lowest tertile (Tertile 1) were determined as Ca [15.45 (9.04, 21.85)], Mg [16.97 (10.59, 23.35)], Zn [7.27 (1.24, 13.30)], Cu [18.50 (12.94, 24.07)], Na [8.35 (1.26, 15.44)], K [15.78 (9.32, 22.23)].

### 3.3 Smooth curve fitting

**Figure 2:**
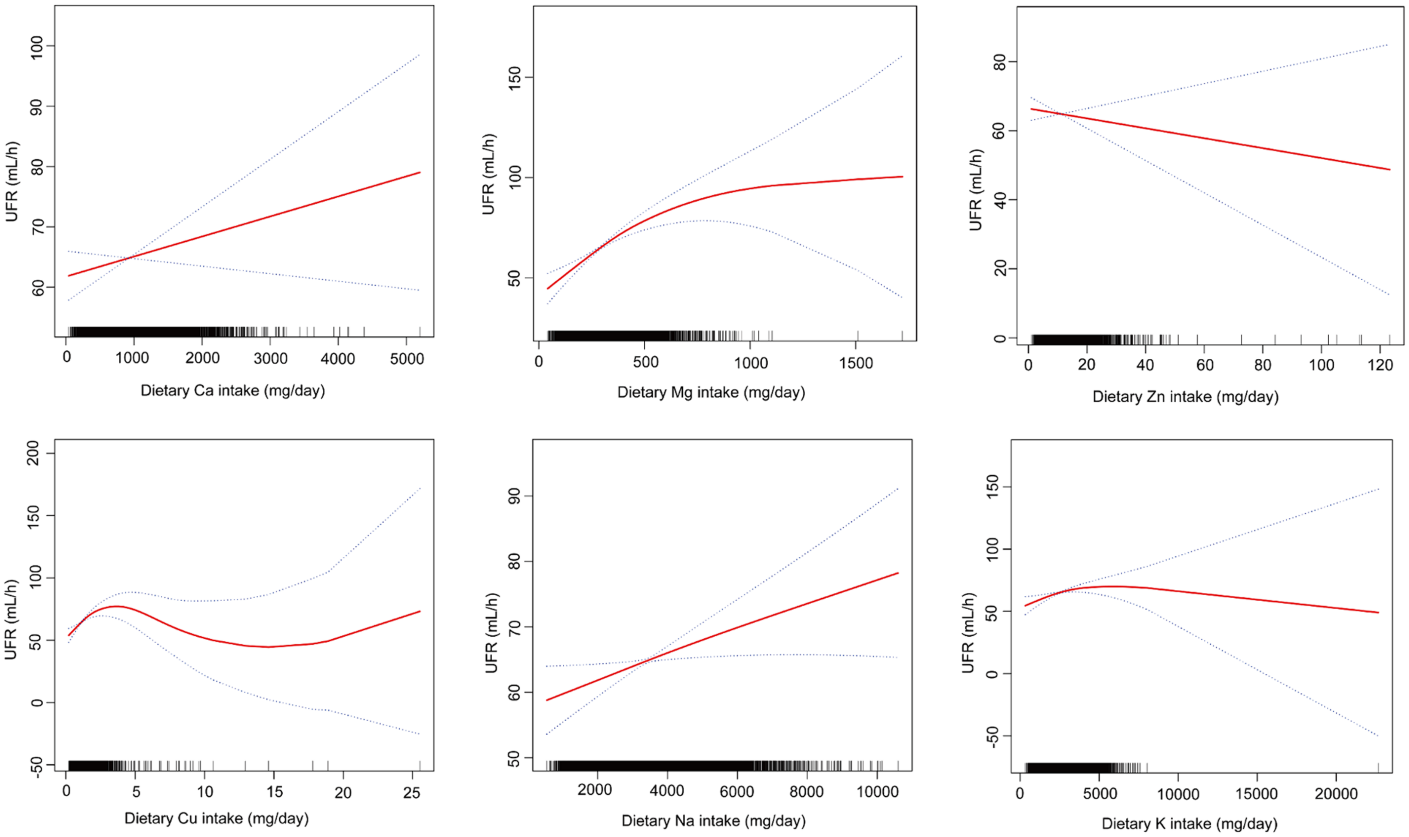
The nonlinear associations between dietary mineral intakes and UFR.

Through smoothing curve fitting, we found a nonlinear relationship between mineral intake (Ca, Mg, Zn, Cu, Na, K) and UFR (Figure 2) and further analyzed the nonlinear relationship for threshold effects (Table 3). The results of the analysis showed that when the mineral intake (Ca, Mg, Zn, Cu, Na, K) was below the inflection point, they were all significantly and positively correlated with UFR (all *p* < 0.05). However, when dietary Mg intake exceeded 353.5 mg/day, or dietary Zn intake exceeded 9.62 mg/day, or dietary Cu intake exceeded 1.35 mg/day, or dietary Na intake exceeded 3,005 mg/day, or dietary K intake exceeded 2979.5 mg/day, none of the effects were significant (all *p* > 0.05). In addition, Ca had a significant effect below the inflection point [0.01 (0.00, 0.02)] and a significant effect above the inflection point [−0.01 (−0.02, −0.00)] in opposite directions (all *p* < 0.05). The *p*-values for LRT were all less than 0.05, indicating that all models showed significant improvement over the simple linear model, supporting the nonlinear relationship between UFR and mineral intake (Ca, Mg, Zn, Cu, Na, K).

**Table 3:** Threshold effect analysis for the relationship between dietary mineral intakes (Ca, Mg, Zn, Cu, Na, K) and UFR using piece-wise linear regression.

Exposure	Ca β (95% CI) *p*-value	Mg β (95% CI) *p*-value	Zn β (95% CI) *p*-value	Cu β (95% CI) *p*-value	Na β (95% CI) *p*-value	K β (95% CI) *p*-value
Model I						
One line effect	0.00 (−0.01, 0.01) 0.8851	0.05 (0.03, 0.08) <0.0001	−0.17 (−0.59, 0.24) 0.4147	1.31 (−1.06, 3.69) 0.2790	0.00 (−0.00, 0.00) 0.5916	0.00 (−0.00, 0.00) 0.1926
Model II						
Turning point (K)	1129.5	353.5	9.62	1.35	3,005	2979.5
<K effect 1	0.01 (0.00, 0.02) 0.0052	0.12 (0.08, 0.16) <0.0001	1.93 (0.76, 3.10) 0.0012	25.16 (16.95, 33.37) <0.0001	0.00 (0.00, 0.01) 0.0452	0.01 (0.00, 0.01) <0.0001
>K effect 2	−0.01 (−0.02, −0.00) 0.0242	0.02 (−0.01, 0.05) 0.2145	−0.37 (−0.81, 0.06) 0.0882	−0.31 (−2.74, 2.12) 0.8026	−0.00 (−0.00, 0.00) 0.8391	−0.00 (−0.01, 0.00) 0.2563
Effect 2–1	−0.02 (−0.03, −0.01) 0.0001	−0.10 (−0.14, −0.06) <0.0001	−2.31 (−3.51, −1.11) 0.0002	−25.47 (−33.86, −17.08) <0.0001	−0.00 (−0.01, −0.00) 0.0439	−0.01 (−0.01, −0.01) <0.0001
LRT	<0.001	<0.001	<0.001	<0.001	0.044	<0.001

Model I: One line effect, Model II: Nonlinear analysis, LRT, log likelihood ratio test; *p*-value < 0.05 means Model II is significantly different from Model I, which indicates a non-linear relationship. Analysis was adjusted for age, gender, race, education level, PIR, BMI, SCr, energy intake, protein intake, carbohydrate intake, total sugars intake, total fat intake, smokers, alcohol drinkers, hypertension, diabetes, history of CVD, and mineral intakes (Ca, P, Mg, Fe, Zn, Cu, Na, K, Se).

### 3.4 Subgroup analyses

We performed subgroup analyses and interaction tests stratified by sex, BMI, hypertension, and diabetes to assess whether associations between mineral intake (Ca, Mg, Zn, Cu, Na, K) and UFR were consistent across the general population and to identify any potentially different population settings (Table 4). The results showed a significant moderating effect of gender on the effect of Mg (*P* for interaction <0.05), which was significantly higher for female [0.09 (0.05, 0.13)] than for male [0.03 (−0.00, 0.07)]. For diabetic patients, Na [0.01 (0.00, 0.02)] showed a significant positive effect, whereas in nondiabetic patients, the effect was not significant, indicating a significant interaction of diabetic status on the effect of Na (*P* for interaction <0.05). In the BMI and hypertension subgroups, the effects of the exposures differed but the interaction did not reach the level of statistical significance (all *p* > 0.05).

**Table 4:** Subgroup analysis of the association between dietary mineral intakes (Ca, Mg, Zn, Cu, Na, K) and UFR.

Exposure	Sex			*P* for interaction
	Male	Female		
Ca	−0.00 (−0.01, 0.01)	0.00 (−0.01, 0.01)		0.7897
Mg	0.03 (−0.00, 0.07)	0.09 (0.05, 0.13)		0.0169
Zn	−0.32 (−0.82, 0.19)	−0.13 (−0.89, 0.63)		0.6900
Cu	2.95 (−0.20, 6.10)	−1.05 (−4.72, 2.62)		0.1040
Na	0.00 (−0.00, 0.00)	−0.00 (−0.01, 0.00)		0.0635
K	−0.00 (−0.00, 0.00)	0.01 (0.00, 0.01)		0.0746

Exposure	BMI				*P* for interaction
	<18.5	≥18.5, <25	≥25, <30	≥30	
Ca	−0.02 (−0.08, 0.05)	−0.00 (−0.01, 0.01)	0.00 (−0.01, 0.01)	−0.00 (−0.01, 0.01)	0.7861
Mg	0.37 (−0.13, 0.88)	0.02 (−0.04, 0.08)	0.04 (0.00, 0.09)	0.06 (0.01, 0.10)	0.3701
Zn	−0.32 (−5.44, 4.79)	0.07 (−0.88, 1.02)	−0.33 (−1.05, 0.38)	−0.24 (−0.88, 0.40)	0.6872
Cu	−32.28 (−106.41, 41.85)	2.56 (−6.18, 11.29)	2.52 (−0.65, 5.69)	−0.11 (−4.17, 3.95)	0.4162
Na	0.01 (−0.02, 0.04)	−0.00 (−0.01, 0.00)	0.00 (−0.00, 0.01)	0.00 (−0.00, 0.01)	0.1374
K	−0.00 (−0.04, 0.03)	0.00 (−0.00, 0.01)	0.01 (0.00, 0.01)	−0.00 (−0.01, 0.00)	0.3566

Exposure	Hypertension			*P* for interaction
	Yes	No		
Ca	0.00 (−0.01, 0.01)	−0.00 (−0.01, 0.01)		0.6383
Mg	0.03 (−0.01, 0.08)	0.06 (0.03, 0.09)		0.2480
Zn	−0.26 (−0.95, 0.42)	−0.14 (−0.67, 0.39)		0.7873
Cu	−0.78 (−4.63, 3.06)	2.46 (−0.51, 5.43)		0.1809
Na	0.00 (−0.00, 0.01)	−0.00 (−0.00, 0.00)		0.1550
K	0.01 (0.00, 0.01)	0.00 (−0.00, 0.00)		0.0926

Exposure	Diabetes			*P* for interaction
	Yes	No		
Ca	−0.00 (−0.02, 0.02)	0.00 (−0.01, 0.01)		0.8869
Mg	0.04 (−0.05, 0.13)	0.05 (0.03, 0.08)		0.7902
Zn	−0.08 (−1.56, 1.41)	−0.20 (−0.64, 0.24)		0.8785
Cu	0.19 (−6.32, 6.70)	1.64 (−0.94, 4.21)		0.6844
Na	0.01 (0.00, 0.02)	−0.00 (−0.00, 0.00)		0.0139
K	0.01 (−0.00, 0.02)	0.00 (−0.00, 0.00)		0.4431

Age, gender, race, education level, PIR, BMI, SCr, energy intake, protein intake, carbohydrate intake, total sugars intake, total fat intake, smokers, alcohol drinkers, hypertension, diabetes, history of CVD, and mineral intakes (Ca, P, Mg, Fe, Zn, Cu, Na, K, Se) were adjusted.

## 4 Discussion

Our study involving 10,229 representative adult NHANES participants shows an association between Mg intake and UFR in a linear regression model for continuous variables. And in the model analysis of tertile categorical variables, we observed a positive association between six mineral intakes (Ca, Mg, Zn, Cu, Na, and K) and UFR. Smoothed curve fitting and threshold effect analysis further support the nonlinear relationship between mineral intake and UFR. Subgroup analyses and interaction tests ensured the reliability and robustness of the findings.

The voiding reflex is influenced by nerve conduction, detrusor function, and bladder outlet (20). Three groups of peripheral nerves innervate the lower urinary tract: the parasympathetic, sympathetic and somatic nervous systems (21). Regarding muscle control, the muscles that primarily affect urination are the detrusor and the pelvic floor muscle groups, which are smooth and skeletal muscles, respectively (20). In terms of bladder outlet, benign prostatic hyperplasia (BPH) is the most common causative factor for bladder outlet obstruction in men (22). Causes of bladder outlet obstruction in women include pelvic organ prolapse, urethral fibrosis or stricture, and detrusor external sphincter dyssynergia (23); pathogenesis includes increased autonomic nervous system activity, detrusor sensitivity, endothelial dysfunction, chronic inflammation and oxidative damage (24).

Ca is the most abundant mineral in the body and needs to be obtained from food. The World Health Organisation recommends a daily Ca intake of 1,000 mg/day for young people (25). The regulation of UFR by Ca is closely linked to the extracellular calcium-sensing receptor (CaSR), which is expressed in human proximal glomerular cells, renal epithelial cells, arterial smooth muscle, and endothelial cells, and can sense and conduct Ca into intracellular signaling pathways (26). CaSR is functionally expressed on all cells of vascular smooth muscle cells and endothelial cells and can regulate changes in vascular tone and arterial blood pressure (27, 28). Activation of the endothelial CaSR allows potassium to be taken up by the myocyte Na/K-ATPase, which helps to reverse vascular myocyte depolarisation and thus vasorelaxation (29). In proximal glomerular cells, dietary Ca uptake mediated through the CaSR reduces the activity of the renin-angiotensin II-aldosterone system (30). In renal epithelial cells, signaling from the basolateral CaSR in the TAL induces natriuresis (31, 32).

Mg is an essential ion for the human body and is a cofactor for many enzymes. We need to consume Mg on a regular basis to maintain normal physiological functions and metabolism in the human body. The United States Food and Nutrition Board recommends a daily intake of 420 mg for men and 320 mg for women (33). Mg can affect urinary function by regulating muscle contraction, vasodilatory tone, muscle and nerve transmission, neuromuscular transmission, and signal transduction (34). Mg decreases small arterial tone and increases the vasodilatory effects of endogenous and exogenous vasodilators, thereby promoting urination. A deficiency in Mg enhances aldosterone synthesis mediated by angiotensin and increases the production of thromboxane and the vasoconstrictor prostaglandin, which leads to reduced urine output (34, 35). Mitochondrial dysfunction also affects bladder smooth muscle contraction, and Mg is required for mitochondria to carry out oxidative phosphorylation (36). Mg can act indirectly as part of the Mg-ATP complex or directly as an enzyme activator (37). Mg is involved in many of the enzyme systems that regulate glucose homeostasis, such as hexokinase and phosphofructokinase. Mg can also influence glucose homeostasis by affecting insulin secretion and cellular uptake of glucose, which can have an effect on UFR (38).

Zn is the second most prevalent trace element in the human body after iron. 85% of Zn is located in muscle and bone (39). Cu is an essential micronutrient for human health and can only be obtained from food (40). Zn is involved in immunity, energy metabolism and antioxidant processes, and is a good antioxidant with protective and therapeutic effects on the central nervous system (41), Zn enhances muscle contraction in vitro (42). A cross-sectional study shows that serum Zn is positively correlated with grip strength (43), while UFR was positively correlated with grip strength (44). Nitric oxide (NO) is thought to be an important neurotransmitter in urethral relaxation and erection and is also involved in the control of bladder afferent nerve activity (21). Zn deficiency disrupts nitric oxide production and increases oxidative stress, leading to endothelial damage (45). A deficiency in dietary Cu significantly attenuates the relaxation and dilation of vascular smooth muscle mediated by nitric oxide (46, 47). Cu deficiency also leads to impaired energy production, abnormal glucose and cholesterol metabolism, and increased oxidative damage (47).

Na is an important part of human nutrition. A study showed that plasma renin activity increased during low Na intake, while brain natriuretic peptide and creatinine clearance decreased, indicating a decrease in intravascular volume (48). Na is involved in maintaining serum electrolyte concentrations, balancing intravascular osmotic pressure, maintaining acid–base balance, and has a role in stimulating muscle contraction (49).

K is an essential nutrient for the maintenance of normal cell function in the body and plays an important role in resting membrane potential and intracellular osmotic pressure (50). K hyperpolarises endothelial cells by stimulating the sodium-potassium pump and activating plasma membrane potassium channels, thereby inducing endothelium-dependent vasodilation (51–53). K can also inhibit the sympathetic nervous system by increasing the uptake of norepinephrine into sympathetic nerve endings, thereby promoting vascular smooth muscle relaxation and increased blood flow (52). Increased K intake also significantly improves endothelial function and increases arterial compliance (54).

Our study found that the effect of dietary magnesium intake on UFR differed significantly between male and female, with female showing higher sensitivity. A clinical trial observed that estrogen levels are significantly higher in female than in male and that estrogen can affect the metabolism of a number of minerals that can enhance magnesium utilization and uptake in soft tissues (55). Differences in habits and basic conditions such as education and marital status between male and female also have an impact (56). In addition, differences in the effect of dietary Mg intake on UFR between men and women may also be related to differences in the structure of the urinary tract in men and women, for example, prostate enlargement in men can cause bladder outlet obstruction, which can affect the UFR (57, 58).

Our study has several advantages over previous studies. First, UFR was used as an outcome variable to assess the effect of dietary mineral intakes on voiding function. Second, we found positive correlations between six dietary mineral intakes (Ca, Mg, Zn, Cu, Na, K) and UFR, providing guidance for preventing and improving urinary function. Finally, this study is based on the NHANES database, which employs a rigorous random sampling process to ensure that our results are sufficiently representative of US adults. However, our study has several limitations. First, due to the cross-sectional analytic design of this study, we were unable to determine a causal relationship between dietary mineral intakes and UFR. Second, despite considering multiple covariates, we were unable to rule out the effects of all potential confounders. Finally, although self-reported dietary intake data are detailed and comprehensive, they may be subject to recall bias and estimation error.

## 5 Conclusion

This study investigated the association between nine dietary minerals (Ca, P, Mg, Fe, Zn, Cu, Na, K, Se) and UFR. The results showed that the intake of six of these minerals (Ca, Mg, Zn, Cu, Na, K) was positively associated with UFR, suggesting that these minerals may have a positive effect on improving urinary function. In particular, the positive correlation between Mg and UFR was more pronounced in women, while the positive correlation between Na and UFR was more prominent in diabetic patients. Although P, Fe, and Se intake did not show significant correlations in the current study, which may require more research to explore their potential association with urinary function. In summary, although these findings provide a preliminary understanding of the relationship between dietary minerals and urinary function, further prospective studies are still necessary to validate these relationships and explore the physiologic mechanisms underlying them.

## Data Availability

Publicly available datasets were analyzed in this study. This data can be found at: www.cdc.gov/nchs/nhanes/.
